# 

**Published:** 1903-12

**Authors:** 


*THE BRISTOL MEDICO-CHIRURGICAL SOCIETY.*

### CONTENTS.

| | PAGE |
|---|---|
| **Some Surprises in my Professional Career.** By J. Paul Bush, C.M.G. ... | 289 |
| **Light Treatment.** By A. J. Harrison, M.B. Lond., and W. Kenneth Wills, M.A., M.B., B.C. Cantab. .. ... ... | 303 |
| **The Dangers of Delay in Ovariotomy.** By T. Carwardine, M.S., M.B. Lond., F.R.C.S. ... ... ... ... ... ... ... ... | 309 |
| **A Case of Infantile Scurvy.** By Bertram M. H. Rogers, M.D., B.Ch., B.A. Oxon. | 318 |
| **Note on Scurvy as a Cause of Hæmaturia in the Infant and in Old Age.** By R. Shingleton Smith, M.D., B.Sc. Lond. | 320 |
| **A Case of Infantile Scurvy or Scurvy-Rickets.** By Christopher Elliott, B.A., M.D. ... ... ... ... ... ... ... | 323 |
| **Trypanosomiasis.** By J. Odery Symes, M.D., D.P.H. Lond. ... ... ... ... ... | 325 |
| **Muscular Atrophy and Sclerodermia.** By J. A. Nixon, M.B., B.C. Cantab., M.R.C.P. Lond. ... ... ... ... ... | 328 |
| **Collargol: A Review of Some of its Clinical Applications, with Experiments on its Antiseptic Action.** By J. M. Fortescue-Brickdale, M.A., M.D. Oxon. ... ... ... ... ... ... ... | 337 |
| **Progress of the Medical Sciences:** | |
| **Medicine.** J. Michell Clarke, M.A., M.D. Cantab. ... ... ... ... ... ... | 345 |
| **Surgery.** James Swain, M.S., M.D. Lond. | 349 |
| **Laryngology.** P. Watson Williams, M.D. Lond. ... ... ... ... ... ... | 354 |
| **Otology.** W. H. Harsant, F.R.C.S. ... | 356 |
| **Reviews of Books** ... ... ... ... ... ... | 358 |
| **Editorial Notes** ... ... ... ... ... ... | 369 |
| **Notes on Preparations for the Sick** ... | 373 |
| **The Library of the Bristol Medico-Chirurgical Society** ... .. ... ... | 377 |
| **Meetings of Societies** ... ... ... ... ... | 379 |
| **Local Medical Notes** ... ... ... ... ... | 383 |

